# Book Review

**Published:** 1933

**Authors:** 


### Reviews of Books

**Idols and Invalids.** By JAMES KEMBLE, Ch.M., F.R.C.S. Pp. 210. London: Methuen & Co. Ltd. 1933. Price 6s.—This is a series of essays concerning the private lives of certain notable persons. The author discusses the lameness of Byron and its effect on his character, and speculates as to the nature of his last illness. He shows how a stone in the bladder ruined Judge Jeffreys's temper and reputation, gives us a catalogue of the mistresses of Louis XV with some notes on the King's health, and explains the patriotic motives underlying Cleopatra's seduction of Cæsar and of Antony. Where he surveys well-documented ground, as in the life of Nelson, his medical opinions are interesting and instructive. With Byron and Queen Anne he is less convincing. Christopher Columbus, Henry VIII, Cesare Borgia, and "Bloody" Queen Mary (among others) he pronounces syphilitic: unless the grounds for this diagnosis, which he suppresses, are far more convincing than those he reveals, we feel that only the laymen among his readers will admit the decision probable, let alone proved. An amusing and readable book.

**A Short History of Surgery.** By Sir D'ARCY POWER, K.B.E., F.R.C.S. Pp. 91. London: John Bale, Sons & Danielsson, Ltd. 1933. Price 3s. 6d.—This series of little books gives brief histories of anatomy, physiology, and other subjects, and into these ninety pages Sir D'Arcy has had to compress the long story of surgery. It is marvellous how much he has included in them, and how fascinating are the sketches he has drawn. The story of the work of Cheselden and still more that of Lister and his followers are full of facts unknown to most of us. Then we have the charming tale of the first great operation under ether in 1846, and that of Pepys the Diarist being taken into a friend's house to be "cut for the stone" in 1658, resolving to give an annual feast in memory of their kindness. Sir D'Arcy has a good word to say for hospital nurses before the Nightingale reforms, believing that they were far more reliable, devoted and skilled than is commonly thought. We are sceptical as to any closing of Roman hospitals in 335 A.D. until more evidence for it can be produced (see p. ii.). There is a trifling misprint of Nusia for Nursia on page 13, but the book is as a whole carefully printed and is well worth reading.

**Diseases of the Heart.** By THOMAS LEWIS, C.B.E., M.D., D.Sc., LL.D. Pp. xx., 297. Illustrated. London : Macmillan & Co. Ltd. 1933. Price 12s. 6d.—In this book the author sets forth his clinical teaching on disease of the heart. No attempt has been made to compile an exhaustive work of reference, but the subject is dealt with in the light of the author's own clinical experience. Much pruning and simplification has been effected, and trivial details and named signs find no place in this work. The statements made are, almost without exception, the result of the writer's own observation. Perhaps as a necessary result of this the style is at times rather dogmatic. It is refreshing to see a complete break-away from the time-honoured custom of dealing with diseases of the heart on an anatomical basis, which has been such a stumbling-block to the teaching of scientific cardiology. As the author rightly insists, a systematic classification of heart disease is hardly possible. Throughout the book stress is laid on the one important factor—the functional capacity of the heart and the means of assessing this capacity. As a result the dominant theme is the importance of the correct evaluation of the patient's symptoms. The book is arranged on novel lines. The earlier chapters deal with the symptoms presented by cardiac patients, then follows a consideration of the various anatomical defects that occur in the heart, and after this the main ætiological types of heart disease are described. Attention is paid to the importance of heart disease in operations, anæsthesia and child bearing, and the last chapter deals with diagnosis, prognosis and treatment in general. This book, which may be regarded as the credo of one who has played an important part in placing cardiology on a scientific basis, can be confidently recommended to all interested in diseases of the heart from whatever point of view.

**Cardiovascular Pain as a Biochemical Problem.** By GORDON LAMBERT, M.D. Pp. xi., 75. Illustrated. London : H. K. Lewis & Co. Ltd. 1933. Price 6s.—This monograph belittles all the theories of the origin of cardiac pain, and suggests that the real explanation will be elucidated by biochemical means. That some change occurs in the blood and in the muscle cells in this condition has been suggested by many authors; but to say that all further efforts by morbid anatomists are useless is to go too far. Much has been attained by these researches, and the cause has been tied down to the coronary circulation. The fact that the starvation of the heart of fresh blood causes the pain is too firmly established to be overthrown, and perhaps microscopic examination of the smaller coronary vessels may yet reveal sufficient data to explain the cases which at present do not appear to fall under the coronary explanation. The illustrations of polygraph and electrocardiographic tracings in the book are singularly lacking in time markings.

**The Common Causes of Chronic Indigestion.** By THOMAS C. HUNT, D.M., M.R.C.P. Pp. vii., 341. Illustrated. London: Baillière, Tindall & Cox. 1933. Price 12s. 6d.—This book can be thoroughly recommended to the practitioner and student of clinical medicine, in that it brings together in a very readable form all the latest information on the approach to and study of the phenomena of chronic indigestion. All sides of the subject are presented with thoroughness and considerable judgment, the author being singularly free from any medical or surgical bias. The close co-operation of the radiologist in the investigation of symptoms is insisted upon; and the volume is illustrated by sixteen beautiful reproductions of the X-ray appearances of various pathological conditions affecting the abdomen. There are two valuable chapters at the end on "Principal Methods of Investigation" and on "Case-Taking and Prescriptions." There is also an excellent bibliography, a good index, and the type, general arrangement and headings are excellent.

**Operative Surgery.** By ALEXANDER MILES, M.D., LL.D., and D. P. D. WILKIE, M.D. Pp. xviii., 590. Illustrated. London: Oxford University Press. 1933. Price 21s.—This book, of about 600 pages, replaces the companion volume to the authors' well-known *Manual of Surgery*. It is intended for those taking classes in operative surgery, candidates for higher surgical examinations, and the "occasional surgeon," but we can assure the authors, and our readers, that not a few professional surgeons will be glad to consult it. The collaboration of no less than sixteen members of the staff of the Edinburgh hospitals has been secured, and surgery of the

L

various regions of the body divided amongst them. The result is excellent. Wide experience and sane judgment speak from every chapter. The directions are clear and definite, and the illustrations really illustrate ; they are neither too many (a common fault in modern books) nor too few. Some very new operations are described, such as lumbar and stellate ganglionectomy, removal of parathyroid tumour, resection of the presacral nerve plexus, and avulsion of the phrenic nerve. We are glad to see a description of Kronlein's operation for orbital tumours, which is missing in some English text-books. There is a particularly good description of splenectomy, and a useful method is offered for the treatment of parotid fistula. We observe that the abdomino-perineal operation for rectal cancer is performed in two stages. Naturally, surgical practice being very individualistic, there are many points we would have written differently. Some very old operations, such as that for the removal of the tongue with part of the mandible, are inserted ; we think it would be well to say that these are now seldom, if ever, performed. In the article on cervical rib "rare" cases are mentioned in which the pressure on the nerve is exerted by the first thoracic rib ; we think these are commoner than a genuine articulated cervical rib, and Adson's treatment by dividing the scalenus anticus might be mentioned. We would personally have included Sloan's method of opening the upper abdomen. Probably a better method of treating pancreatic cyst than that described is to drain it into the stomach. We do not approve the advice to infold perforations of a gastric ulcer ; it is enough to approximate the edges and cover with a flap of omentum. We do not like drainage tubes in the pelvis for this condition (having many years ago lost two patients from intestinal obstruction due to the tube), but greatly prefer a strip of corrugated rubber dam. We are glad to see that suction is advised instead of swabbing out. However, the authors have just as much right to their own opinion, and altogether have produced a very excellent manual, smaller and cheaper than good books on operative surgery usually are.

**Radiologic Maxims.** By HAROLD SWANBERG, M.D. Pp. 127. Quincy, Illinois, U.S.A.: Radiological Review Publishing Co. 1932. Price $1.50.—This book consists of reprints from the monthly "Radiological Maxims" of the *Radiological Review*. Even for these no originality is claimed : there is a complete absence of useful information, no attempt at classification, and considerable repetition. A quotation from Pope appears on the title-page: "Half our knowledge we must snatch, not take." The line is appropriately chosen.

**A Shorter Orthopædic Surgery.** By R. BROOKE, M.S. Pp. viii., 150. Illustrated. Bristol: John Wright & Sons, Ltd. 1932. Price 10s. 6d.—This book has been specially written for the use of masseuses, house surgeons, and those who require a short outline of the methods and treatment usually adopted in orthopædic surgery. Mr. Brooke has adopted a regional method for his descriptions, and although this has its advantages, particularly for those whose medical knowledge is limited, it has also serious drawbacks. As an example one may take tuberculous disease. Of this there is no general description, and so the account of its manifestations in various regions entails needless reduplication. The same criticism applies to rickets, and poliomyelitis receives but scant attention. The author appears to be fonder of the use of plaster in the treatment of tuberculous bones and joints than are many. He even describes the ambulatory treatment of sacro-iliac disease, which most would deem to be unjustifiable. The ways of surgeons vary vastly, but few would find much with which to quarrel in this little book, and it can safely be commended to those for whom it is particularly intended.

**The Principles of Treatment of Muscles and Joints by Graduated Muscular Contractions.** By MORTON SMART, C.V.O., D.S.O., M.D. Pp. xv., 217. London: Oxford University Press. 1933. Price 15s.—How nice it is to come across a book which really makes one think. After reading this work by Dr. Smart one cannot help giving one's ideas a thorough overhaul. Is our treatment of fractures and sprains all that can be desired? Will our routine of splinting, massage and so forth bear the cold light of reason? How can we avoid pain, stiff joints, and chronic œdema? These are some of the many questions which the author sets out to answer. His reasoning appears to be sound, and few of his readers but will try out some of his methods. Personal experience of the methods of Lucas Championnière leads the reviewer to approach with favour any method which will permit of the early movement of muscles. This the author accomplishes by causing muscular contractions to take place by means of a surging faradic current, the power of which is kept under personal control throughout the treatment. The muscles may thus be caused to contract so feebly that no movement of the joint occurs, yet the muscle is prevented from becoming adherent; the circulation in the muscle and neighbouring parts is improved; and the muscle itself is kept from wasting. The details of treatment are preceded by a full account of the physiology and pathology of muscle and of the principles upon which treatment is based. This is a book which deserves to be widely read.

**Clinical Ophthalmology for House Surgeons and Students.** By J. MILES BICKERTON, M.A., B.Ch., and L. H. SAVIN, M.D. Pp. vii., 158. Illustrated. London: H. K. Lewis & Co. Ltd. 1933. Price 7s. 6d.—The authors have attempted to teach the uninitiated a correct technique in the various methods of examining the eye, and to deal adequately, if briefly, with most of its diseases. For so small a book they have achieved their purpose with a surprising degree of success. The subject-matter is clearly set out and easily understood, and the diagrams and illustrations are adequate and helpful. Minor operations are explained fully, there is a useful chapter on "Ophthalmic Formulæ," and an appendix, with full illustrations, of instruments required for ophthalmic operations. As a companion to work in the Out-patient Department and Wards, or as a practical guide for the General Practitioner, this book is admirable.

**A Doctor to a Mother.** By EARDLEY HOLLAND, M.D., R. C. JEWESBURY, M.D., and WILFRED SHELDON, M.D. Pp. 96. London: Edward Arnold. 1933. Price 1s. 6d.—This intensely practical booklet is written in words easily understandable by the lay mother, for whom it is intended. The first part deals with the mother's care of herself from the onset of pregnancy to the onset of labour, stressing the need for ante-natal care. Next come chapters on breast and artificial feeding and tables and instruction for the feeding of infants after weaning, up to four years of age. This last is a very useful addition and so often omitted in similar books. The remaining chapters are devoted to diseases and the minor ailments of infants, their treatment and prevention, ending with a talk on the milestones in the child's mental and bodily development. The whole book is a model, giving the mother sensible advice but telling her when it is necessary to consult a doctor, without frightening her with alarming symptoms. It is a book which one can recommend to all mothers.

**A Standard Classified Nomenclature of Disease.** By H. B. LOGIE, M.D. Pp. xvii., 702. New York: Commonwealth Fund. 1933. Price 21s.—The object of this book is to promote a uniform standard classification of disease among American hospitals and medical men. It represents the considered judgment of all the most important medical associations of the United States. " Each disease is described in terms of the tissue or organ where it is principally manifested, and in etiological terms." A numerical code system is incorporated in which the whole history of any given case can be expressed in a series of digits: with great convenience for statistical and similar enthusiasts. Those for whose comfort and use the volume is intended will best know how far it achieves its object.

**Surgical Operations.** By E. W. HEY GROVES, M.D., B.Sc., M.S. Third Edition. Pp. viii., 263. Illustrated. London: Oxford University Press. 1933. Price 18s.—This text-book makes a most admirable, lucid, and comprehensive compendium of surgery for nurses and young doctors. It has been brought right up to date; describing, for example, modern methods, such as Kirschner's extension apparatus for the treatment of fractures and the use of radium in the treatment of cancer of the tongue. The clarity of the descriptions and sketches and the valuable appendix illustrating all the ordinary surgical instruments are meritorious features of the book. Possibly the time is shortly coming when the surgery of the eye and other specialized work will be better excluded entirely, leaving room for additional details of operations of general surgery. One or two points need attention and amplification. The old-fashioned Barker's apparatus for anæsthesia should be replaced by illustrations and descriptions of the Pitkin type of instrument; and a little more might usefully be said on the *modus operandi* of spinal anæsthesia, for a good deal of the vital supervision of the cases falls into the hands of the nurse. A simple description is given of the method of combating the danger of the inspiration of vomited matter into the lungs during the operation for strangulated hernia. This might well be supplemented by informing the reader how, by spinal anæsthesia, the danger can be entirely avoided. This book fills a very valuable place in the surgical education of the nursing profession.

